# Determinants of Vitamin D Levels in Italian Children and Adolescents: A Longitudinal Evaluation of Cholecalciferol Supplementation versus the Improvement of Factors Influencing 25(OH)D Status 

**DOI:** 10.1155/2014/583039

**Published:** 2014-11-11

**Authors:** Stefano Stagi, Paola Pelosi, Massimo Strano, Giovanni Poggi, Cristina Manoni, Maurizio de Martino, Salvatore Seminara

**Affiliations:** ^1^Health Sciences Department, University of Florence, Anna Meyer Children's University Hospital, 50139 Florence, Italy; ^2^Pediatric Endocrinology Unit, Health Sciences Department, University of Florence, Anna Meyer Children's University Hospital, Viale Pieraccini 24, 50139 Florence, Italy; ^3^Pediatric Unit, Mugello Hospital, Borgo San Lorenzo, 50032 Florence, Italy

## Abstract

*Objective.* This paper aims to assess 25(OH)D levels in Italian children and adolescents identifying risk factors for 25(OH)D deficiency and to evaluate whether a normal 25(OH)D value can be restored in 25(OH)D-deficient patients. *Methods.* We evaluated 25(OH)D levels in 679 Italian children and adolescents (≤10, 11–20, 21–30, and >30 ng/mL were defined as severe deficiency, deficiency, insufficiency, and sufficiency, resp.). Of these, 365 25(OH)D-deficient were followed up for 1 year; 205 were treated with cholecalciferol (Arm A: 400 I.U.) and 160 by improving the environmental variables influencing 25(OH)D levels (Arm B). *Results.* At cross-sectional evaluation, 11.3% showed sufficiency, 30.0% insufficiency, and 58.7% 25(OH)D deficiency. Mean 25(OH)D was 19.08 ± 8.44 ng/mL. At the enrollment time (*T*_0_), no difference was found between Arms A and B with respect to distribution and 25(OH)D levels. At end time (*T*_1_) 26.0% (29.7% in Arm A versus 20.6% in Arm B) showed sufficiency, 38.4% (42.0% versus 34.4%) insufficiency, and 35.6% (28.3% versus 45.0%) 25(OH)D deficiency. Mean 25(OH)D level was 23.71 ± 6.83 ng/mL. *Conclusions.* Neither changes of lifestyle nor 400 I.U. cholecalciferol supplementation alone appears to be sufficient to restore adequate 25(OH)D levels.

## 1. Introduction

Vitamin D status is highly variable among European countries, largely due to variations in exposure to sunshine, dietary intake of vitamin D, and the use of supplements [1, 2]. Vitamin D can be synthesized endogenously, and factors affecting its cutaneous synthesis include age, season, latitude, time of day, skin pigmentation, amount of skin exposed, and the use of sunscreen [2].

Vitamin D status is normally defined according to the serum concentration of 25-hydroxyvitamin D [25(OH)D] [3]. Usually, vitamin D deficiency is defined as having a serum 25(OH)D concentration lower than 50 nmol/L (20 ng/mL) [3]. Nevertheless, an evaluation of satisfactory levels of vitamin D in healthy children has not yet been reported by adequate studies [3–5].

Based on the current literature, the prevalence of 25(OH)D deficiency varies from 2 to 30% in adults [2, 6]. However, in a small-scale Italian study, more than 80% of children had insufficient or deficient levels of 25(OH)D [7]. Two larger studies providing transversal data have confirmed that 25(OH)D deficiencies are very common among Italian children [8–10].

An adequate 25(OH)D status is very important because 25(OH)D deficiency is a risk factor for several chronic diseases in addition to having classic deleterious effects on bone. Vitamin D deficiency can cause secondary hyperparathyroidism, which may lead to high bone turnover and bone loss and ultimately increase the risk of fractures [2–5]. Emerging evidence suggests that vitamin D also plays an important role in immune system regulation. Vitamin D receptors are found on several immune cells, and vitamin D metabolites appear to modulate T-cell proliferation and dendritic cell function [11, 12]. Vitamin D deficiency may also be a risk factor for the development of autoimmune and other chronic diseases [3, 11, 13, 14], loss of muscle mass, and muscle weakness [15]. Finally, a number of studies have also suggested that vitamin D may confer protection against diabetes mellitus Type 1, hypertension, multiple sclerosis, and cancer [16]. Thus, vitamin D insufficiency may have important health consequences not only because of the vitamin's role in the maintenance of normal bone mass turnover but also because of its role as an immunoregulatory agent. The serum 25(OH)D level is the most commonly measured indicator of vitamin D status because it reflects dietary intake from vitamins D2 and D3 together with cutaneous synthesis of vitamin D3 [17].

Although data are not available regarding the required vitamin D intake in children, a clinical report from the American Academy of Pediatrics (AAP) recommended a daily intake of 400 I.U./day of vitamin D for all infants, children, and adolescents [18], in contrast with a previous recommendation of 200 I.U. (5 *μ*g)/day [19].

To date, few studies have assessed vitamin D status among Italian children [7–10], and, to our knowledge, there have been no longitudinal studies. The available data indicate that, due to lifestyle changes, the main source of vitamin D, via synthesis in the skin from cholesterol after exposure to UV-B light, has been significantly reduced due to the large amount of time spent indoors [18, 19].

Thus, the purpose of this study was to assess serum 25(OH)D levels in a large cohort of children and adolescents living in the Italian province of Florence in the Tuscany region of Italy (latitude 44°N) and to identify risk factors for vitamin D deficiency in different age groups. This study also aimed to evaluate whether a normal 25(OH)D value can be restored in 25(OH)D-deficient patients with a daily supplement of 400 I.U. of cholecalciferol or by improving the factors influencing 25(OH)D status.

## 2. Patients and Methods

We consecutively evaluated 679 Caucasian children and adolescents (326 males and 353 females, aged 2.1–17.9) from Mugello, an area of Tuscany, and Florence (central Italy), Italy. All of the subjects were selected by random cluster sampling of the subjects who were seen at the Pediatric Unit of Mugello's Hospital of Borgo San Lorenzo or the Endocrine Paediatric Unit of the Anna Meyer Children's University Hospital between September 2010 and December 2013 for routine control visits. The Hospital Ethics Committees of Anna Meyer Children's University Hospital and Mugello's Hospital approved the study, which was conducted in accordance with the Declaration of Helsinki guidelines. Written informed consent was obtained from the parents/guardians of all of the subjects.

### 2.1. Study Design

The present study aimed first to assess cross-sectionally the serum 25(OH)D levels in a very large cohort of Italian children and adolescents and to identify risk factors for vitamin D deficiency in these subjects.

For the cross-sectional evaluation, the inclusion criteria were as follows: patients older than 2 and younger than 18. The exclusion criteria were as follows: a recent history of travelling to warmer, sunnier areas prior the study, use of calcium or vitamin D supplements or any drugs affecting calcium or vitamin D metabolism within the past six months, or a positive history of primary hyperparathyroidism or other skeletal diseases, malabsorptive disorders, or neurological or renal diseases.

Based on the protocol, this study also included a 12-month (*T*_0_ − *T*_1_) controlled longitudinal study comparing the abilities of vitamin D supplementation and the improvement of factors influencing 25(OH)D status to ameliorate 25(OH)D deficiencies.

**Figure 1:**
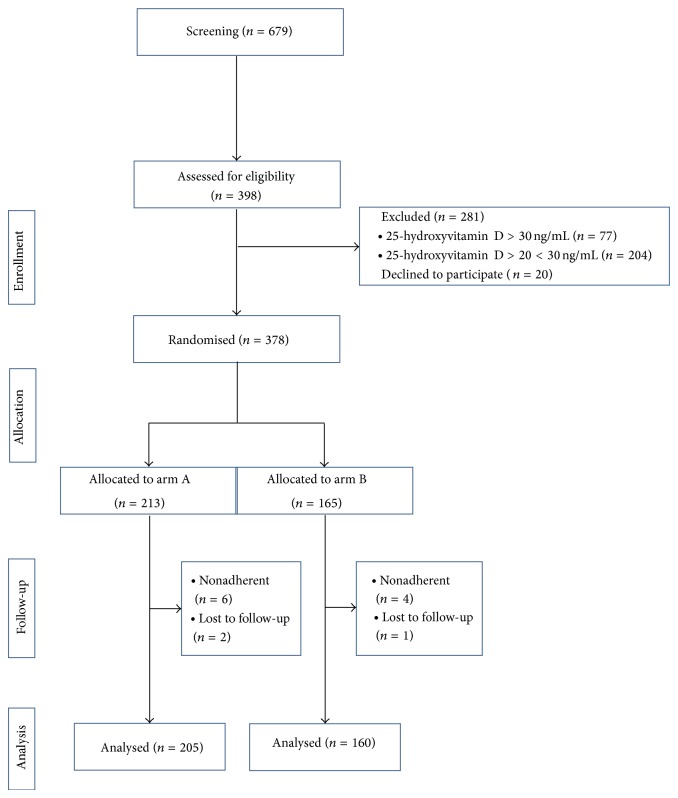
Summary of patient flow diagram. Individuals with 25(OH)D deficiency. Patients in the Arm A group received 400 I.U. of vitamin D3 daily during the study, whereas patients in the Arm B group worked to improve factors that influence 25(OH)D status but did not receive vitamin D3 supplements.

The patients found to have a 25(OH)D deficiency during the cross-sectional evaluation were recruited for the longitudinal study (Figure 1). Of the 679 Caucasian subjects initially included in the cross-sectional evaluation, 398 children and adolescents (186 males and 212 females, aged 3.1 to 18.9) having 25(OH)D levels below 20 ng/mL were enrolled in the interventional study (Figure 1). The mean time elapsed between the first (*T*_0_) and the second (*T*_1_) determinations was 12.1 months (range: 11.8–12.3 months).

These patients were randomly divided into two groups. Randomization was performed using a computer-generated random number table with 1.3 : 1 randomization within strata defined by gender and age.

The patients in the first group, Arm A, were treated with cholecalciferol 400 I.U. (10 *μ*g)/daily. Of these subjects (225 subjects, 105 males and 120 females), 12 declined the consent to participate in the study, and 8 dropped out due to noncompliance, lack of follow-up, and so forth. In all, 205 of these individuals (101 males and 104 females) completed the interventional study and constituted the subjects of Arm A.

The patients in the second group, Arm B, did not receive cholecalciferol supplements, but they were instructed to improve variables influencing 25(OH)D levels, including diet, milk intake, hours spent outdoors, sun exposure, and use of sunscreen. Of these subjects (173 subjects: 81 males and 92 females), 8 declined the consent to participate in the study, and 5 dropped out due to noncompliance or lack of follow-up. The remaining 160 individuals (71 males and 89 females) completed the interventional study and were the subjects of Arm B.

### 2.2. Sample Size Calculation

The number of subjects required to compare the abilities of the two treatment arms to attain serum 25(OH)D concentrations of at least 30 ng/mL and to detect a change of at least 1.5 nmol/L in serum 25(OH)D levels between groups was calculated with a significance level of 5% and a power of 90%. The aim was to include at least a 1.3 : 1 ratio due to a higher withdrawal expected in Arm A.

### 2.3. Study Protocol

During the cross-sectional evaluation and the interventional study (*T*_0_ and *T*_1_), the clinical and demographic data were collected from the subjects, including height, weight, body mass index (BMI), pubertal stage, time dedicated to outdoor physical activity, sunlight exposure, and use of sunscreen. Furthermore, nutrient diaries were recorded for each subject based on their medical charts and standardized interviews. For both the cross-sectional and interventional (*T*_0_ and *T*_1_) studies, the subjects were also divided into two age groups: children (2–12 years) and adolescents (older than 12 years).

During the cross-sectional evaluation, the dietary intakes of calcium and vitamin D were assessed through a standardized interview with the parents, recording birth weight, type of feeding during the first year (breast, formula milk, or mixed), the mother's use of vitamin supplements during pregnancy, and the child's use of vitamin D supplements [4, 5]. Depending on the age of the child, the parent or guardian and the child were also interviewed about the frequency of consumption (daily, weekly, and monthly) of each food item with the use of food models, portion booklets, or serving containers to assist in estimating serving size. Nutrient analyses were obtained from the Food Composition Database for Epidemiological Studies in Italy (Banca Dati di Composizione degli Alimenti per Studi Epidemiologici in Italia—BDA). A daily intake of more than 200 mL per day of cow's milk (median content of vitamin D was 40 I.U./L) was considered adequate [4, 5, 20].

During the same interview, we recorded the hours/week of outdoor physical activity (consequently subdividing our study group into low level outdoor physical activity (≤2 hours/day) and high level (>2 hours/day)) and categorized the activity using a physical activity questioner, as previously described [20]. Outdoor exposure was quantified both from questions regarding each child's average number of daily outdoor hours during each season and from a prospective daily-time-activity diary completed by caregivers during the study.

During these interviews, the amount of sun exposure was also calculated and evaluated in terms of the number of days of significant exposure to sunlight before the cross-sectional evaluation and during the period of the study or for the interventional study, the summer previous to study enrolment. At our latitude (44°N), cutaneous synthesis of vitamin D takes place only during the summer months (May–September) [4, 5, 21]. We defined significant exposure to sunlight as the exposure of arms and legs for 15 minutes between 10 a.m. and 3 p.m. without the application of sunscreen [22], identifying the following three categories: poor (<15 days), moderate (15–30 days), and good (≥30 days) sun exposure. Sunscreen use was also evaluated and defined as regular (use of a sunscreen with a sun protection factor (SPF) ≥ 15 applied at least 30 minutes before sun exposure and subsequently every 2 hours) or irregular [23].

During the study, all of the subjects underwent laboratory tests to measure their plasma 25(OH)D levels as well as serum calcium, phosphate, bone-specific alkaline phosphatase (B-ALP), and parathyroid hormone (PTH) levels.

### 2.4. Vitamin D Status

The serum 25(OH)D levels were stratified according to the following brackets: ≤10, 11–20, 21–30, and >30 ng/mL and defined as severe deficiency, deficiency, insufficiency, and sufficiency, respectively, according to previously established guidelines for bone health (in the absence of a consensus regarding appropriate levels for endocrine and extraendocrine health) [3–5].

To evaluate seasonal variations, we divided the year into four seasons. However, although solar winter is typically defined as the period from November to February, to create larger, more significant statistical groups, we defined winter as the period from November to May and summer as that from June to October because human insolation to UVB radiation is negligible between November and April [21].

### 2.5. Vitamin D Intervention (*T*_0_ and *T*_1_)

All of the subjects of Arm A were treated with 400 I.U. (10 *μ*g) of cholecalciferol administered orally once daily. The cholecalciferol supplement was purchased from Abiogen Pharma S.p.A. (Pisa, Italy), and the drops contained 250 I.U. (6.25 *μ*g) of cholecalciferol. The dosage was based on the recommendation of the American Academy of Pediatrics for all infants, children, and adolescents [18, 19].

For Arm A, written instructions were provided at the onset of the study, and compliance was evaluated by a written questionnaire completed by the parents. Compliance was further verified by e-mails and/or telephone interviews performed by a study nurse and by the bottle count of cholecalciferol performed at the end of the study period.

### 2.6. Implementation of the Variables Influencing 25(OH)D Levels (*T*_0_ and *T*_1_)

For Arm B, after evaluating the questionnaires about calcium and vitamin D dietary intake, outdoor physical activity levels, sun exposure, and the use of sunscreen, the parents and children were informed of how to safely increase vitamin D levels; this information provided them with new knowledge and had the potential to change their attitudes and behaviors.

To eliminate differences in knowledge, at *T*_0_, all of the Arm B families participating in the study received written and oral information about the importance of vitamin D in the health of children and adults and the amount of vitamin D required for good health (Dietary Reference Intake or DRI). To optimize the consumption of nutrients naturally rich in vitamin D, such as fish, fatty fish, eggs, milk, and dairy products, the parents received information about the major dietary sources of vitamin D. This information was provided using the Food Composition Database for Epidemiological Studies in Italy (Banca Dati di Composizione degli Alimenti per Studi Epidemiologici in Italia - BDA).

The equal importance of a certain amount of sun exposure gained through outdoor activities was also explained.

For Arm B, written instructions were provided at the onset of the study and compliance was evaluated by a written questionnaire completed by the parents. Compliance was further verified by e-mails and/or telephone interviews performed by a study nurse.

### 2.7. Measurements

Each subject's weight was measured with electric scales to the nearest 0.1 kg, and the height was measured with a stadiometer. The body mass index (BMI) was calculated using the following formula: BMI = weight (kg)/height (m)^2^. Tanner's pubertal stage was determined at baseline and at each visit. The age-related reference values for height, bone age, and BMI obtained from large sample numbers of Italian children and currently used in Italy were used in the study [24]. As described previously [24], height and BMI were normalized for chronological age by conversion to standard deviation scores (SDS), which were calculated according to the following formula:

 Patient value − mean of age-related reference value/standard deviation of the age-related reference value [25].

The pubertal staging was performed according to the criteria of Tanner and Whitehouse [27], using an orchidometer for the boys. Blood samples were obtained from each study participant after an overnight fast. The plasma concentrations of calcium, phosphate, and B-ALP were determined following routine biochemical laboratory protocols. Serum 25(OH)D and PTH levels were determined by chemiluminescence enzyme-labeled immunometric assays using an IMMULITE 2000 Systems analyzer (Siemens, Gwynedd, UK). The intra- and interassays CVs were < 5% and <8% and <8% and <10%, respectively. All samples were measured in one laboratory that takes part in and meets the performance targets for the vitamin D external quality assessment scheme (DEQAS).

### 2.8. Statistical Analyses

The statistical analyses were performed using SPSSX software (SPSSX Inc, Chicago, IL, USA). The characteristics of the study population were described through frequency distributions for categorical variables and through means and standard deviations (SDs), medians, and ranges for continuous variables, depending on whether the data were normally distributed. The differences between patient groups and controls were assessed using Student's *t*-test or the Mann-Whitney *U* test, depending on the distribution of the analyzed variable. The chi-square test and Fisher's exact test were used as appropriate to examine associations between dichotomous variables. Intergroup comparisons for parameters were conducted using analysis of variance (ANOVA) or repeated-measures analysis of covariance (ANCOVA), as appropriate.

Spearman's and/or Pearson's correlation tests were used to determine the correlation coefficients. A multiple stepwise regression was performed to investigate factors associated with insufficient vitamin D status after adjusting for potential confounders. *P* values < 0.05 were considered to be statistically significant.

## 3. Results

### 3.1. Cross-Sectional Evaluation

The main cross-sectional results are shown in Table 1. Among the 679 subjects, 482 (71.0%) were found to have a normal weight, 168 (24.7%) were overweight, and 29 (4.3%) subjects were obese. Divided by sex, 241 of 353 females (68.3%) had a normal weight, 91 (25.8%) were overweight, and 21 (5.9%) were obese, whereas 238 of 326 males (73.0%) had a normal weight, 77 (23.6%) were overweight, and 11 (3.4%) were obese.

Of the 679 subjects, 453 (66.7%) were prepubertal, whereas 226 (33.3%) were pubertal. Divided by sex, 237 of the 353 females (67.1%) were prepubertal and 116 (32.9%) were pubertal, whereas 217 of the 326 males (66.5%) were prepubertal and 109 (33.5%) were pubertal.

With respect to 25(OH)D levels, 77 of 679 (11.3%) subjects had sufficient levels, 204 (30.0%) had insufficient levels, 259 (38.2%) showed deficient levels, and 139 (20.5%) had a severe deficiency.

Among all the patients, the mean level of 25(OH)D was 19.08 ± 8.44 ng/mL. Among those with sufficient 25(OH)D levels, the mean concentration was 33.91 ± 3.51 ng/mL. In contrast, the mean concentration was 24.58 ± 4.78 ng/mL for those with insufficient levels, 15.16 ± 2.71 ng/mL for those with deficient levels, and 7.56 ± 1.64 ng/mL for those with a very deficient level.

Subdividing the group into children and adolescents, the children presented with a mean 25(OH)D level of 20.26 ± 8.43 ng/mL, whereas the mean level in adolescents was 11.74 ± 5.78 ng/mL (*P* < 0.0005).

Finally, subdividing the group according to BMI, we found that subjects with a normal weight had significantly higher 25(OH)D levels (25.66 ± 7.32 ng/mL) than overweight subjects (17.13 ± 7.87 ng/mL, *P* < 0.0005), obese subjects (12.02 ± 5.67 ng/mL, *P* < 0.0005), or obese and overweight subjects combined (15.17 ± 7.34 ng/mL, *P* < 0.0005).

**Figure 2:**
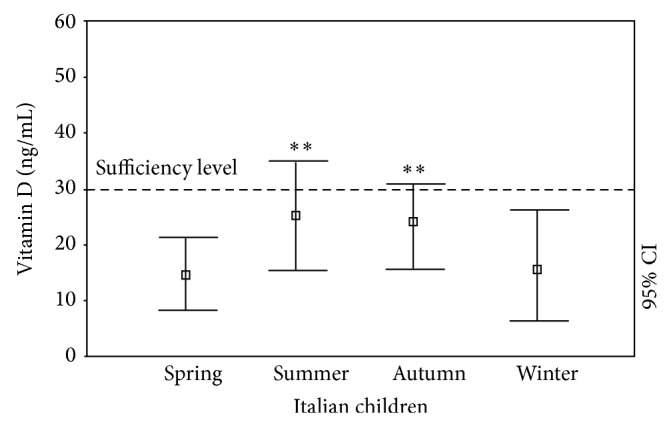
25(OH)D levels (ng/mL) in Italian children and adolescents during different seasons in the cross-sectional evaluation (versus spring value). ^*^ *P* < 0.05; ^**^ *P* < 0.005; ^***^ *P* < 0.001.

There was a marked seasonal effect on 25(OH)D levels. In fact, we found that the Italian children in our study had deficient mean 25(OH)D levels in spring (15.0 ± 6.43 ng/mL). Although the levels were significantly higher in summer (25.72 ± 8.60 ng/mL, *P* < 0.005) and autumn (23.25 ± 6.58, *P* < 0.005 versus spring values; *P* = NS versus summer values), they were still not sufficient. In winter, the 25(OH) D levels were also significantly lower than those observed in summer and fall (15.57 ± 9.17 ng/mL, *P* < 0.005 versus summer and autumn values) and similar to the levels observed in spring (*P* = NS versus spring value) (Figure 2). If the year is divided into only two periods (winter as November–May and summer as June–October), the mean 25(OH)D level was 23.76 ± 7.58 ng/mL in summer and 15.96 ± 8.65 ng/mL in winter (*P* < 0.0005).

Evaluating the effect of hours spent outdoors on 25(OH)D levels, we showed a significant difference between the group spending ≤ 2 hours/day in outdoor physical activity (14.04 ± 7.49 ng/mL) and the group spending > 2 hours/day in outdoor physical activity (23.76 ± 7.58, *P* < 0.005).

The mean intake of vitamin D was 179.3 ± 43.9 I.U. per day. There was no significant difference in the vitamin D intake between females and males, children and adolescents, or normal weight, overweight, and obese subjects. However, the children with appropriate daily intake of cow's milk showed significantly higher 25(OH)D levels (24.41 ± 7.82 ng/mL) than the children who consumed no or only small quantities of cow's milk per day (17.60 ± 8.74 ng/mL, *P* < 0.005).

The subjects with low sun exposure showed significantly reduced 25(OH)D levels (13.47 ± 7.54 ng/mL) than the subjects with moderate (17.99 ± 8.87 ng/mL; *P* < 0.0001) and good (25.23 ± 9.88 ng/mL; *P* < 0.0001) sun exposure. Finally, the subjects who regularly used sunscreen exhibited significantly lower 25(OH)D levels (15.44 ± 7.65 ng/mL) than subjects who did not use sunscreen regularly (24.65 ± 9.99 ng/mL; *P* < 0.0001).

Evaluating all of the subjects, we found that 108 of 679 patients (15.9%; 51 males, 57 females) had PTH levels above the normal range; the mean PTH level was 51.13 ± 36.46 pg/mL. Of the 679 patients, 172 (25.3%; 90 males, 82 females) had high B-ALP levels; the mean B-ALP level was 129.3 ± 36.7 U/L.

Evaluating the correlations between 25(OH)D levels and age, sex, BMI, cow milk consumption (mL/day), outdoor physical activity (hours/week), use of sunscreen, PTH, calcium, phosphorus, B-ALP, birth weight, type of feeding during the first year of life, mother's use of vitamin supplementation during pregnancy, child's past use of vitamin D supplementation, and daily intake of cow milk, we found that 25(OH)D levels correlated with age (*r* = −0.56, *P* < 0.0001), BMI (*r* = −0.67, *P* < 0.0001), cow's milk consumption (*r* = 0.53, *P* < 0.005), hours/week of outdoor physical activity (*r* = 0.76, *P* < 0.0001), sunscreen use (*r* = −0.63, *P* < 0.005), sun exposure (*r* = 0.69, *P* < 0.0001), PTH levels (*r* = −0.59, *P* < 0.001), calcium levels (*r* = 0.39, *P* < 0.005), B-ALP (*r* = −0.47, *P* < 0.005), maternal feeding during the first year of life (*r* = 0.29, *P* < 0.005), and the child's past use of vitamin D supplementation (*r* = 0.45, *P* < 0.0005) but not with sex, phosphorus levels, birth weight, or the mother's use of vitamin supplementation during pregnancy.

### 3.2. One Year Interventional Study: Enrollment Time or* T*_0_

The main results of the one-year interventional study at *T*_0_ are shown in Table 2. Of the 378 total subjects included in the interventional study, 272 (72.0%) were of normal weight, 91 (24.1%) were overweight, and 15 (4.0%) were obese (Table 2). Similar results were found when the subjects were divided into Arms A and B. In Arm A, 153/213 (71.8%) were of normal weight, 51/213 (23.9%) were overweight, and 9/213 (4.2%) were obese, whereas in Arm B, 119/165 (72.1%) were of normal weight, 40/165 (24.3%) were overweight, and 6/165 (3.6%) were obese. We did not find significant differences compared with the results of the cross-sectional evaluation.

Furthermore, we did not find any significant differences between Arm A and Arm B with respect to the prepubertal/pubertal ratio (in Arm A, 144/213 subjects (67.6%) were prepubertal, whereas 69 (32.4%) were pubertal, and in Arm B, 111/165 (67.3%) were prepubertal, whereas 54 (32.8%) were pubertal). No significant differences between the interventional study and the cross-sectional evaluation were found with respect to the prepubertal/pubertal ratio.

At *T*_0_, of the 378 subjects, 243 (64.3%) had deficient 25(OH)D levels, and 135 (35.7%) exhibited a severe deficiency. In Arm A, 139/213 (65.3%) showed 25(OH)D deficient levels, and 74 (34.7%) had a severe deficiency. In Arm B, 104/165 subjects (63.0%) demonstrated deficient 25(OH)D levels, and 61 (37.0%) had a severe deficiency. No significant difference was found between Arms A and B with respect to the distribution of 25(OH)D levels.

Among the total number of patients participating in the study, the mean level of 25(OH)D at *T*_0_ was 12.53 ± 4.64. There was no significant difference in the mean 25(OH)D levels at *T*_0_ between Arm A (12.42 ± 4.58 ng/mL) and Arm B (12.64 ± 4.72 ng/mL). When we subdivided the subjects of the two arms into children and adolescents, for children, mean 25(OH)D levels of 14.04 ± 4.45 and 14.56 ± 4.22 ng/mL were found in Arms A and B, respectively. For adolescents, the mean 25(OH)D levels were significantly reduced compared with those observed for the children (9.87 ± 4.19 and 9.96 ± 4.65 ng/mL in adolescents in Arms A and B; *P* < 0.0001 resp.).

As seen in the cross-sectional evaluation, when we subdivided the Arms A and B subjects according to BMI, we found that children and adolescents of normal weight presented with significantly higher 25(OH)D levels (Arm A: 14.52 ± 4.21 ng/mL; Arm B: 14.34 ± 4.14 ng/mL) than the overweight (Arm A: 11.01 ± 4.39 ng/mL; Arm B: 11.38 ± 4.23 ng/mL; *P* < 0.0001 versus Arm A and Arm B normal weights) and obese (Arm A: 8.69 ± 3.14 ng/mL; Arm B: 8.54 ± 3.57 ng/mL; *P* < 0.0001 versus Arms A and B normal weight) subjects in the respective arms. Furthermore, the 25(OH)D levels of obese subjects in Arms A and B were significantly lower than the levels observed in the overweight subjects in Arms A and B, respectively, (*P* < 0.05).

**Figure 3:**
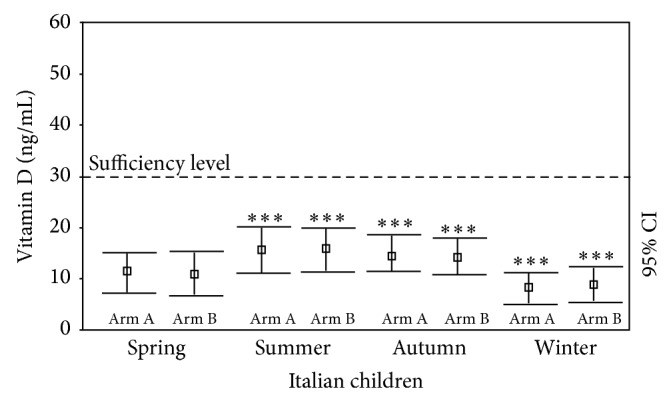
Seasonal variation in 25(OH)D levels (ng/mL) in Italian children and adolescents with 25(OH)D deficiency at baseline (*T*_0_) evaluation (versus spring value) in Arm A and Arm B. ^*^ *P* < 0.05; ^**^ *P* < 0.005; ^***^ *P* < 0.001.

As seen in the cross-sectional evaluation, there was a seasonal effect on 25OHD levels at *T*_0_ evaluation. In fact, we found that the 25(OH)D levels were significantly reduced in spring (11.4 ± 3.76 ng/mL) in 25(OH)D-deficient subjects, without significant differences between Arm A and Arm B (Figure 3). However, 25(OH)D levels were higher in summer (16.37 ± 4.34 ng/mL, *P* < 0.0001 versus spring values) whereas significantly reduced in autumn (14.25 ± 4.07 ng/mL, *P* < 0.005 versus spring values; *P* < 0.005 versus summer values) and winter (8.48 ± 2.94, *P* < 0.0001 versus other seasons) (Figure 3), without significant differences between Arm A and Arm B. If the year is divided into only two periods (winter as November–May and summer as June–October), the mean 25(OH)D level was 14.39 ± 4.58 ng/mL in summer and 9.92 ± 3.97 ng/mL in winter (*P* < 0.0001).

With respect to the number of hours spent outdoors, we found no significant differences between the subjects of Arms A and B (25% of Arm A subjects and 27% of Arm B subjects spent no time outdoors, 46% of Arm A subjects and 49% of Arm B subjects spent < 2 hours per week outdoors, and 29% of Arm A subjects and 24% of Arm B subjects spent > 2 hours per week outdoors). However, we demonstrated a significant difference in 25(OH)D levels between the groups that spent ≤ 2 hours/day in outdoor physical activity (Arm A: 10.20 ± 3.78 ng/mL, Arm B: 10.14 ± 3.67 ng/mL) and those in the respective arms that spent > 2 hours/day in outdoor physical activity (Arm A: 13.80 ± 5.02 ng/mL, Arm B: 13.64 ± 4.67 ng/mL, *P* < 0.0005 for both comparisons).

No significant difference between Arms A and B was found in the mean intake of vitamin D (Arm A: 177.3 ± 45.1 I.U. per day, Arm B: 170.1 ± 47.8 I.U. per day). As found in the cross-sectional evaluation, no significant differences were found in vitamin D intake between females and males, children and adolescents, or normal weight, overweight, and obese subjects. However, in both arms, we found that subjects with appropriate intake of cow's milk had significantly higher 25(OH)D levels (Arm A: 13.99 ± 4.53 ng/mL, Arm B 13.71 ± 4.11 ng/mL) than the children who consumed little or no cow's milk per day (Arm A: 11.18 ± 4.22 ng/mL, Arm B: 11.06 ± 3.99 ng/mL, *P* < 0.0001 for both comparisons).

In both Arms A and B, a low level of sun exposure was associated with significantly reduced 25(OH)D levels (Arm A: 9.82 ± 4.57 ng/mL, Arm B: 9.96 ± 3.89 ng/mL) compared with levels observed in subjects with a moderate (Arm A: 13.23 ± 4.54 ng/mL, Arm B: 13.17 ± 4.11 ng/mL, *P* < 0.0005 for both comparisons) or good (Arm A: 15.34 ± 4.43 ng/mL, Arm B: 15.45 ± 3.98 ng/mL, *P* < 0.0001 for both comparisons) sun exposure; no statistical differences were found between Arms A and B. Regarding the use of sunscreen, we confirmed that the subjects who regularly used sunscreen had significantly lower 25(OH)D levels (Arm A: 10.14 ± 4.65 ng/mL, Arm B: 10.29 ± 4.58 ng/mL) than those with nonregular use of sunscreen (Arm A: 14.65 ± 3.99 ng/mL, Arm B: 14.69 ± 4.88 ng/mL, *P* < 0.0001 for both comparisons).

We found no significant differences in the frequency of hyperparathyroidism between Arm A (47/213, 22.1%) and Arm B (38/165, 23.0%). The mean PTH level was 52.88 ± 33.41 pg/mL in Arm A and 54.54 ± 37.67 pg/mL in Arm B; these levels were not significantly different. In Arm A, 69/213 subjects (32.4%) had high B-ALP levels, as did 57/165 subjects (34.5%) in Arm B. The mean B-ALP level was 131.1 ± 37.3 U/L in Arm A and 128.0 ± 34.9 U/L in Arm B.

Evaluating the correlations among 25(OH)D levels and age, sex, BMI, cow's milk consumption (mL/day), outdoor physical activity (hours/week), use of sunscreen, sun exposure, PTH, calcium, phosphorus, B-ALP, birth weight, type of feeding during the first year of life, mother's use of vitamin supplementation during pregnancy, and child's past use of vitamin D supplementation, we found a significant correlation between 25(OH)D levels and age (*r* = −0.53, *P* < 0.0001), BMI (*r* = −0.61, *P* < 0.0001), cow's milk consumption (*r* = 0.58, *P* < 0.005), hours/week of outdoor physical activity (*r* = 0.64, *P* < 0.0001), use of sunscreen (*r* = −0.60, *P* < 0.005), PTH levels (*r* = −0.51, *P* < 0.001), calcium levels (*r* = 0.47, *P* < 0.005), B-ALP (*r* = −0.53, *P* < 0.001), type of feeding during the first year of life (*r* = 0.38, *P* < 0.005), and the child's past use of vitamin D supplementation (*r* = 0.49, *P* < 0.0005). No correlation was found between 25(OH)D levels and sex, phosphorus levels, birth weight, or the mother's use of vitamin supplementation during pregnancy.

### 3.3. One-Year Interventional Study: End Time or *T*_1_

The main results of the interventional study at *T*_1_ are shown in Table 2. At *T*_1_, of the 365 patients who completed the study, 287 (78.6%) were of normal weight, 61 (16.7%) were overweight, and 17 (4.7%) were obese. These findings were not significantly different than those observed at *T*_0_ (72.0% of normal weight, 24.1% overweight, and 4.0% obese). Moreover, no difference in the percentages of subjects who were of normal weight, overweight, and obese at *T*_1_ and *T*_0_ was found when the two arms were considered individually (Arm A: 147/205 (71.7%) were normal weight, 49/205 (23.9%) overweight, and 9/205 (4.4%) were obese, Arm B: 117/160 (73.1%) normal weight, 39/160 (24.4%) overweight, and 4/160 (2.5%) obese).

At *T*_1_, 236/365 (64.7%) subjects were prepubertal, whereas 129 (35.3%) were pubertal (*P* = NS when the results were compared with those obtained at *T*_0_). No significant differences in the percentages of prepubertal and pubertal patients were observed between the arms at *T*_1_ (Arm A: 137/205 (66.8%) subjects were prepubertal and 68/205 (33.2%) were pubertal; Arm B: 106/160 (66.2%) were prepubertal and 54/165 (33.8%) were pubertal).

At *T*_1_, 95/365 (26.0% versus 0.0% at *T*_0_; *P* < 0.0001) subjects had sufficient 25(OH)D levels, 140 (38.4% versus 0.0% at *T*_0_; *P* < 0.0001) had insufficient levels, 80 (21.9% versus 64.3% at *T*_0_; *P* < 0.0001) showed deficient levels, and 50 (13.7% versus 35.7% at *T*_0_; *P* < 0.0001) demonstrated a severe deficiency. At *T*_1_, of the 205 subjects in Arm A, 61 (29.7% versus 0.0% at *T*_0_; *P* < 0.0001) had sufficient 25(OH)D levels, 86 (42.0% versus 0.0% at *T*_0_; *P* < 0.0001) had insufficient levels, 43 (21.0% versus 65.3% at *T*_0_, *P* < 0.0001) showed deficient levels, and 15 (7.3% versus 34.7% at *T*_0_; *P* < 0.0001) had a severe deficiency. In contrast, of the 160 subjects in Arm B, 33 (20.6% versus 0.0% at *T*_0_; *P* < 0.0001) had reached sufficient 25(OH)D levels, 55 (34.4% versus 0.0% at *T*_0_; *P* < 0.0001) had insufficient levels, 37 (23.1% versus 63.0% at *T*_0_; *P* < 0.0001) showed deficient levels, and 35 (21.9% versus 37.0% at *T*_0_; *P* < 0.005) had a severe deficiency. At *T*_1_, a significantly greater percentage of subjects in Arm A had a more sufficient level of 25(OH)D than those in Arm B (29.7% versus 21.2%; *P* < 0.05), and a significantly reduced percentage of subjects in Arm A were considered 25(OH)D-deficient compared to Arm B (28.3% versus 45%; *P* < 0.005). Only 7.3% of subjects in Arm A had a severe 25(OH)D deficiency at *T*_1_, whereas 21.9% of the subjects in Arm B were severely deficient (*P* < 0.0001).

At *T*_1_, the mean level of 25(OH)D of all the patients in the study was 23.71 ± 6.83 ng/mL, significantly higher than that observed at *T*_0_ (12.53 ± 4.64 ng/mL; *P* < 0.0001). More specifically, at *T*_1_, the mean 25(OH)D concentration was 34.78 ± 4.04 ng/mL in the subjects with 25(OH)D sufficiency, 24.89 ± 3.56 ng/mL in those who had insufficient 25(OH)D levels, 19.49 ± 3.77 ng/mL (versus 15.23 ± 3.07 ng/mL at *T*_0_, *P* < 0.0001) among those who had deficient 25(OH)D levels, and 9.23 ± 1.87 ng/mL (versus 7.42 ± 1.78 ng/mL at *T*_0_, *P* < 0.0001) among those who had very deficient 25(OH)D levels.

When the subjects were divided in the two arms, those in Arm A showed significantly higher 25(OH)D levels (25.03 ± 6.84 ng/mL) than those in Arm B (21.35 ± 7.30 ng/mL, *P* < 0.0001). In particular, the 25(OH)D-sufficient subjects in Arm A had higher mean 25(OH)D levels than the sufficient subjects in Arm B (35.87 ± 3.86 ng/mL versus 32.58 ± 5.39 ng/mL; *P* < 0.005).

Subdividing the group into children and adolescents, at *T*_1_, children presented a mean 25(OH)D level that was significantly higher than that found at *T*_0_ (23.45 ± 7.76 ng/mL versus 14.34 ± 4.33 ng/mL, *P* < 0.0001). Similar results were observed when each arm was analyzed separately (Arm A: 24.89 ± 7.54 at *T*_1_ versus 14.04 ± 4.45 at *T*_0_, Arm B: 22.77 ± 7.99 at *T*_1_ versus 14.56 ± 4.22 ng/mL at *T*_0_, *P* < 0.0001 for both comparisons). Adolescents also presented with significantly higher 25(OH)D levels at *T*_1_ compared with that observed at *T*_0_ (14.89 ± 4.86 ng/mL versus 9.93 ± 4.38 ng/mL, *P* < 0.0001). Similar results were found when each arm was analyzed individually (Arm A: 16.00 ± 5.03 at *T*_1_ versus 9.87 ± 4.19 at *T*_0_; Arm B: 14.11 ± 5.99 at *T*_1_ versus 9.96 ± 4.65 ng/mL at *T*_0_, *P* < 0.0001 for both comparisons).

When the subjects were subdivided according to BMI, at *T*_1_, the combined children and adolescents of normal weight showed significantly higher 25(OH)D levels (23.99 ± 5.66 ng/mL) than overweight subjects (17.13 ± 7.87 ng/mL, *P* < 0.0005) or obese individuals (12.02 ± 5.67 ng/mL, *P* < 0.0001). These differences were maintained when Arms A and B were analyzed separately (Arm A: normal weight subjects (25.42 ± 6.67 ng/mL), overweight subjects (21.12 ± 6.23 ng/mL; *P* < 0.005), and obese individuals (14.12 ± 5.43 ng/mL; *P* < 0.0001); Arm B: normal weight subjects (22.12 ± 6.54 ng/mL), overweight subjects (16.34 ± 6.01 ng/mL; *P* < 0.0005), and obese subjects (11.22 ± 6.02 ng/mL; *P* < 0.0001)).

**Figure 4:**
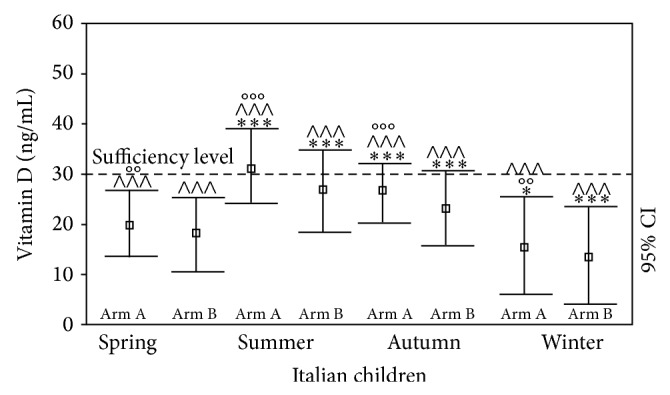
Seasonal variation in 25(OH)D levels (ng/mL) in Italian children and adolescents with 25(OH)D deficiency after cholecalciferol supplementation (Arm A) or implementation of the factors influencing 25(OH)D status (Arm B) at *T*_1_ (versus spring value and versus *T*_0_ evaluation). ^*^ *P* < 0.05; ^**^ *P* < 0.005; ^***^ *P* < 0.001 versus spring value. ^∧^ *P* < 0.05; ^∧∧^ *P* < 0.005; ^∧∧∧^ *P* < 0.001 versus *T*_0_ evaluation. °*P* < 0.05; °°*P* < 0.005; °°°*P* < 0.001 (Arm A versus Arm B values).

Despite the efforts to boost 25(OH)D levels in this study, a seasonal effect on 25(OH)D levels persisted (Figure 4). Although a significant improvement in 25(OH)D was observed in most seasons, the 25(OH)D levels in the Italian subjects remained insufficient in spring (18.90 ± 7.43 at *T*_1_ versus 15.11 ± 6.55 ng/mL at cross-sectional evaluation, *P* < 0.0001), winter (16.93 ± 9.70 at *T*_1_ versus 15.41 ± 9.03 ng/mL at cross-sectional evaluation,* P* = NS), autumn (26.01 ± 6.54 at *T*_1_ versus 23.25 ± 6.58 ng/mL at cross-sectional evaluation, *P* < 0.005), and summer (29.27 ± 8.01 at *T*_1_ versus 25.72 ± 8.60 ng/mL at cross-sectional evaluation, *P* < 0.0001). Similar results were observed when the two arms were analyzed individually, although the subjects in Arm A reached normal (sufficient) 25(OH)D values in summer (32.11 ± 7.23 versus 27.03 ± 8.87 ng/mL in Arm B subjects, *P* < 0.0001).

The subjects in Arm B showed a significant increase in the number of hours spent outdoors per week (2.59 ± 0.86 at *T*_1_ versus 1.87 ± 0.65 at *T*_0_, *P* < 0.0001). Our results demonstrated that there were significant differences in 25(OH)D levels between the subgroup of Arm B spending ≤ 2 hours/day in outdoor physical activity (15.29 ± 7.11 ng/mL) and those spending > 2 hours/day in outdoor physical activity (26.49 ± 6.33 ng/mL, *P* < 0.005), with a significant improvement in 25(OH)D levels only achieved in the subgroup spending > 2 hours/day outdoors. In contrast, in Arm A, there was no significant difference between the 25(OH)D level of the subgroup spending ≤ 2 hours/day outdoors (13.66 ± 7.78 ng/mL) and that of the subgroup spending > 2 hours/day outdoors (18.79 ± 8.11 ng/mL).

Furthermore, a significant improvement in the mean intake of vitamin D was observed in Arm B (264 ± 53.7 I.U per day at *T*_1_ versus 170.1 ± 47.8 I.U. per day at *T*_0_, *P* < 0.0001), whereas no improvement in vitamin D intake was observed in Arm A (191.4 ± 53.7 I.U. per day at *T*_1_). In particular, there was an increase in the percentage of subjects in Arm B who consumed a normal amount of cow's milk over the course of the study (47.9% versus 37.2%, *P* < 0.05). However, at *T*_1_, only Arm B subjects who consumed a normal amount of cow's milk per day showed significantly higher 25(OH)D levels than those who consumed little or no cow's milk per day (26.79 ± 8.65 ng/mL versus 17.17 ± 8.18 ng/mL, *P* < 0.0001). In Arm A, at *T*_1_, no difference was found in the 25(OH)D levels of those who consumed a normal amount of cow's milk and those who consumed little or no cow's milk (20.78 ± 8.33 ng/mL versus 16.59 ± 8.15 ng/mL).

In Arm B, we demonstrated an increase in the length of the sun exposure, with an increase in the percentage of subjects with moderate sun exposure (48.8% versus 32.7%, *P* < 0.005). There was a significant increase in 25(OH)D levels compared with those measured at *T*_0_ for individuals with low sun exposure (13.12 ± 8.71 versus 9.90 ± 4.41 ng/mL, *P* < 0.0001), moderate exposure (16.77 ± 6.43 versus 13.19 ± 4.32 ng/mL, *P* < 0.0001), and good exposure (24.55 ± 6.11 versus 15.39 ± 4.29 ng/mL, *P* < 0.005). In Arm A, the 25(OH)D levels were significantly reduced with respect to those detected in the Arm B subjects for each sun exposure subgroup (11.88 ± 8.12 ng/mL in those with low sun exposure, 14.66 ± 9.01 ng/mL in those with moderate exposure, and 21.65 ± 8.84 in those with good exposure, *P* < 0.0001 for all comparisons).

At *T*_1_, there was no significant difference in the regular use of sunscreens in the subjects of Arm B. We did confirm that the subjects who regularly used sunscreen had significantly lower 25(OH)D levels than nonregular sunscreen users (17.86 ± 7.84 ng/mL versus 26.45 ± 8.56 ng/mL, *P* < 0.0001). There was a significant difference between the 25(OH)D levels of Arm A regular sunscreen users and nonregular users (17.13 ± 8.01 ng/mL versus 26.98 ± 8.77 ng/mL, *P* < 0.0001) and between the 25(OH)D levels of Arm B regular sunscreen users and nonregular users (18.46 ± 7.55 ng/mL versus 26.03 ± 8.79, *P* < 0.0001).

When all of the subjects were included, we demonstrated at *T*_1_ a significant reduction of the percentage of subjects with hyperparathyroidism (83/365 patients (22.7%), 41 males and 42 females). The mean PTH level was 47.87 ± 31.12 pg/mL (versus 53.27 ± 34.78 pg/mL at *T*_0_; *P* < 0.05). Arm A subjects showed significantly reduced PTH levels compared with Arm B subjects (43.01 ± 28.81 pg/mL versus 49.74 ± 33.71 pg/mL, *P* < 0.05). After the interventional study, the mean B-ALP level was 112.5 ± 30.6 U/L in Arm A and 121.7 ± 32.5 U/L in Arm B (*P* < 0.05).

In our evaluation of potential correlations between 25(OH)D levels and factors potentially affecting 25(OH)D levels at *T*_1_ (age, sex, BMI, cow's milk consumption (mL/day), outdoor physical activity (hours/week), use of sunscreens, sun exposure, PTH, calcium, phosphorus, birth weight, type of feeding during the first year of life, mother's use of vitamin supplementation during pregnancy, and child's past use of vitamin D supplementation), we demonstrated that 25(OH)D levels correlated with age (*r* = −0.57, *P* < 0.005), BMI (*r* = −0.63, *P* < 0.005), cow's milk consumption (*r* = 0.49, *P* < 0.005), outdoor physical activity (*r* = 0.66, *P* < 0.0001), use of sunscreen (*r* = −0.49, *P* < 0.005), sun exposure (*r* = 0.53, *P* < 0.005), PTH levels (*r* = −0.61, *P* < 0.001), and calcium levels (*r* = 0.44, *P* < 0.005), and B-ALP (*r* = −0.57, *P* < 0.001). No correlation was found between 25(OH)D levels and sex, phosphorus levels, birth weight, type of feeding during the first year of life, mother's use of vitamin supplementation during pregnancy, and child's past use of vitamin D supplementation at *T*_1_.

## 4. Discussion

Our study provides data on the 25(OH)D status in a large sample of children and adolescents in Mugello, an area of Tuscany, Italy. The extent of the deficiency in most of the children and adolescents was surprising, confirming a recent paper of Vierucci et al. [8, 9]. In fact, our data shows a very high prevalence of vitamin D deficiency (near 56%) in different age groups. Moreover, if we also include individuals with an insufficiency, the percentage rises to 88.6%, very similar to the findings of Vierucci et al. (79.5%) in a different area of Tuscany [8] and Bellone et al. in a series of northern Italian children (71.7%) [10].

As expected, our data also demonstrated a strong seasonal influence on vitamin D status, as observed in some studies performed in countries at northern latitudes and in different age groups [21, 22], whereas other studies reported counterintuitive results at different latitudes [23], probably due to differences in climate. This seasonal effect is probably related to the reduced exposure of children to sunlight and the reduced vitamin D production in the skin during the winter months. In winter, children spend more time indoors, and when they do go out, the amount of skin exposed to the sun is less than that during warmer months. As a consequence, little vitamin D is produced in the skin [10]. Furthermore, at our latitude, sun exposure is only effective in promoting vitamin D activation from May through September [21]. Thus, the amount of vitamin D produced and stored October through April is probably not sufficient to guarantee an optimal vitamin D status in unsupplemented healthy children and adolescents, especially if other factors limit summer sun exposure.

However, the high percentage of vitamin D-deficient and insufficient children in this paper as well as that reported by Vierucci et al. and Bellone et al. [8–10] compared with that reported in previous papers [24–28] may be the result of changes in the lifestyles of children and adolescents, with many hours each day spent in indoor activities, such as school, watching television, or playing games. However, the use of sunscreen is also an important factor influencing this very high percentage of children with vitamin D inadequacy.

In our study, the children had higher 25(OH)D levels than adolescents, most likely due to more outdoors activity, milk intake, and dietary intake of calcium and vitamin D, confirming the results of several previous studies [22, 29–31].

Thus, the presence of such a high percentage of subjects with vitamin D deficiency or insufficiency may be considered a “disease” of modern society. In fact, modern lifestyles and food habits have probably accentuated the predisposing environmental conditions. The implications of this major inadequacy in the health of children and adolescents need to be better defined.

Interestingly, our study demonstrated that neither lifestyle changes nor supplementation with 400 I.U. (10 *μ*g)/day of cholecalciferol appears to be individually sufficient to restore adequate levels of vitamin D in all Italian children and adolescents. Although many studies have investigated vitamin D supplementation in healthy adults [32, 33] as well as adults [34–36], children, and adolescents [13, 37, 38] with chronic diseases, there are few studies in the literature which focused on the effect of regular daily vitamin D supplementation in healthy children and adolescents [39, 40].

In 323 black and white children who randomly received 0, 400, 1000, 2000, or 4000 IU/d of oral vitamin D3 for 12 weeks during the winter, increases in vitamin D concentrations ranging from −10 nmol/L for the placebo to 76 nmol/L for 4000 I.U. supplementation were demonstrated [39]. The authors reported that supplementation with 400 I.U./day was sufficient to maintain adequate wintertime 25(OH)D concentrations in healthy black but not white children [39]. However, in another study evaluating 150 healthy children with baseline serum 25(OH)D levels < 30 ng/mL, after 4 months of nutritional intervention, the subjects supplemented with 400 I.U/d of vitamin D all reached normal vitamin D levels [40]. In contrast, of the subjects who received dietary counseling alone, only one achieved a normal vitamin D concentration [40]. These results are in contradiction with our data, in which we identified a considerable group of subjects (almost 15% of those included in the study) with severe vitamin D deficiency who did not respond to lifestyle changes or cholecalciferol supplementation. Although we demonstrated an improvement in the other classes of 25(OH)D levels (sufficient, insufficient, and deficient levels), over 35% of the subjects remained 25(OH)D-deficient, and over 73% of the patients had levels below the sufficiency level after vitamin D supplementation or implementation of lifestyle changes to improve vitamin D levels. Specifically, after implementing an improved diet, higher intake of milk, and increased hours spent outdoors, although we showed an improvement of nearly 25% in 25(OH)D levels, 21% of the subjects were still severely 25(OH)D-deficient. Among the available data of Italian children and adolescents [4, 5, 8–10], it is interesting to note that one study reported that 54.8% of 93 subjects had serum 25(OH)D levels < 20 ng/mL, despite the fact that 33% of the subjects were receiving vitamin D supplementation at the time of evaluation [41].

Other studies have demonstrated the importance of lifestyle changes in improving 25(OH)D levels. In fact, one study demonstrated that two cups (500 mL) of cow's milk per day was sufficient to maintain 25(OH)D levels above 75 nmol/L [42]. Our results demonstrated that subjects who consumed more than 250 mL of milk/day had improved 25(OH)D levels and also identified an inverse association between the intake of cow's milk and 25(OH)D deficiency, concordant with other studies, indicating the importance of a correct diet in reducing a 25(OH)D deficit [43]. A study of the dietetic habits of our children and adolescents has demonstrated an increased consumption of alternatives to milk (such as soy milk and cereals) that provide little vitamin D and interfere with calcium uptake [44].

The association of a higher risk of 25(OH)D deficit and limited time spent outdoors reflects another modern change of the lifestyle of children and adolescents and, as reported in other studies [22], may indicate the need for cholecalciferol supplementation to help maintain adequate levels of vitamin D in Italian children and adolescents. The promotion of an active outdoor lifestyle may counteract the vitamin D deficiency epidemic, as suggested by a study of Saudi children that demonstrated that children who are physically active have higher levels of vitamin D than those who are less active, despite having the same amount of sun exposure [45]. These findings are also confirmed in the report about 414 girls by Dahifar et al., in which the mean serum 25(OH)D concentration increased to 14.4± ng/mL with sunlight exposure [46].

Our data demonstrated that the season of blood withdrawal is a significant predictor of vitamin D status, with winter and spring being the seasons associated with lower median 25(OH)D levels, in the range of deficiency. This finding is in accordance with other reports [9, 33, 37, 47, 48] and stresses the importance of the promotion of an active outdoor lifestyle, especially from May to September.

These findings are of particular concern because of both the well-established and newly identified target effects of vitamin D (bone accrual and health immunomodulatory function in decreasing the risk of many chronic illnesses, including common cancers, autoimmune diseases, infectious diseases, and cardiovascular disease), stressing the importance of maintaining appropriate 25(OH)D levels [11–16]. Our data seem to confirm and suggest that, individually, lifestyle changes or vitamin D supplementation is not sufficient to ensure sufficient 25(OH)D levels. Consequently, a global intervention or an increase of daily supplementation by cholecalciferol should be considered.

One limitation of our study was that it is an interventional open study rather than a randomized controlled trial. Another limitation was the heterogeneous group of subjects (obese, overweight, and normal weight children and adolescents); however, this limitation was also a benefit because it allowed us to exhaustively evaluate all of the factors involved in vitamin D status. The large number of children and adolescents enrolled in our study was a strength. The lack of vitamin D supplementation at baseline in all subjects was an additional strength, allowing us to evaluate a large number of vitamin D status predictors and differentiate between the effects of vitamin D supplementation and other factors influencing vitamin D status.

## 5. Conclusions

In conclusion, deficient or insufficient vitamin D serum levels were found in most of the Italian children and adolescents sampled in this study. The presence of inadequate 25(OH)D levels represents a complex problem that reflects lifestyle changes, increasingly poor dietary habits, sunscreen use, and an increase in the obesity rate in the population. Our findings suggest that neither changes in lifestyles nor or supplementation with 400 I.U. (10 *μ*g)/day of cholecalciferol appears to be individually sufficient to restore adequate levels of vitamin D. Thus, daily supplementation with more than 400 I.U. (10 *μ*g) should be considered.

## Figures and Tables

**Table 1:** Main data of the cross-sectional evaluation of Italian children and adolescents.

Number of subjects (female : male)	679 (353 : 326)
Age, yrs. (median, range)	8.7 (2.1–17.9)
Height (SDS)	−0.1 ± 1.5
BMI (SDS)	0.5 ± 1.1
Prepubertal/pubertal ratio	4532 : 226
Serum 25(OH)D status	
sufficiency (%)	11.3
insufficiency (%)	30.0
deficiency (%)	38.2
severe deficiency (%)	20.5
Serum 25(OH)D level (ng/mL)	19.08 ± 8.44
children	20.26 ± 8.43
adolescents	11.74 ± 5.78
normal weight	25.66 ± 7.32
overweight	17.13 ± 7.87
obese	12.02 ± 5.67
Dietary intake of vitamin D, IU/day	179.3 ± 43.9
Serum 25(OH)D (ng/mL)	
≤2 hrs/day in outdoor activity	14.04 ± 7.49
>2 hrs/day in outdoor activity	23.76 ± 7.58
normal cow's milk consumption/day	24.41 ± 7.82
reduced cow's milk consumption/day	17.60 ± 8.74
Parathyroid hormone (pg/mL)	51.13 ± 36.46

**Table 2:** Comparison of 25(OH)D determinants in Arms A and B of the interventional study.

	Arm A *T*_0_	Arm A *T*_1_	Arm B *T*_0_	Arm B *T*_1_
Number of subjects (female : male)	213 (200 : 178)	205 (200 : 178)	165 (193 : 172)	160 (193 : 172)
Age, yrs. (median, range)	8.8 (3.1–18.9)	8.8 (3.1–18.9)	9.8 (3.2–19.9)	9.8 (3.2–19.9)
Height (SDS)	0.0 ± 1.5	0.0 ± 1.4	−0.1 ± 1.6	0.0 ± 1.5
BMI (SDS)	0.5 ± 1.1	0.5 ± 1.1	0.5 ± 1.0	0.5 ± 1.0
Prepubertal/pubertal ratio	255 : 123	255 : 123	236 : 129	236 : 129
Serum 25(OH)D status				
sufficiency (%)	—	29.7^∧∧∧^	—	20.6^°°°∗^
insufficiency (%)	—	42.0^∧∧∧^	—	34.4°°°
deficiency (%)	65.3^§§§^	21.0^∧∧∧^	63.0^§§§^	23.1°°°
severe deficiency (%)	34.7^§^	7.3^∧∧∧^	37.0^§§§^	21.9^°°°∗∗^
Serum 25(OH)D (ng/mL)	12.42 ± 4.58^§§§^	25.03 ± 6.84^∧∧∧§§§^	12.64 ± 4.72	21.35 ± 7.30^°°°∗∗∗^
children	14.04 ± 4.45^§§§^	24.89 ± 7.54^∧∧∧§§§^	14.56 ± 4.22	22.77 ± 7.99^°°°∗∗^
adolescents	9.87 ± 4.19^§§§^	16.00 ± 5.03^∧∧∧§§§^	9.96 ± 4.65	14.11 ± 5.99^°°°∗^
normal weight	14.52 ± 4.21^§§§^	25.42 ± 6.67^∧∧∧§§§^	14.34 ± 4.14	22.12 ± 6.54^°°°∗∗^
overweight	11.01 ± 4.39^§§§^	21.12 ± 6.23^∧∧∧§§§^	11.38 ± 4.23	16.34 ± 6.01^°∗∗^
obese	8.69 ± 3.14	14.12 ± 5.43^∧§^	8.54 ± 3.57	11.22 ± 6.02
Dietary intake of vitamin D, IU/day	177.3 ± 45.1^§§§^	191.4 ± 53.7^∧∧§§§^	170.1 ± 47.8	264 ± 53.7^°°°∗∗∗^
Serum 25(OH)D (ng/mL)				
normal milk consumption	13.99 ± 4.53^§§§^	20.78 ± 8.33^∧∧∧§§§^	13.71 ± 4.11	26.79 ± 8.65^°°°∗∗∗^
reduced milk consumption	11.18 ± 4.22^§§§^	16.59 ± 8.15^∧∧∧§§§^	11.06 ± 3.99	17.17 ± 8.18°°°
Serum 25(OH)D (ng/mL)				
≤2 hrs outdoor activity/day	10.20 ± 3.78^§§§^	13.66 ± 7.78^∧∧∧§§^	10.14 ± 3.67	15.29 ± 7.11°°°
2 hrs outdoor activity/day	13.80 ± 5.02^§§§^	18.79 ± 8.11^∧∧∧§§§^	13.64 ± 4.67	26.49 ± 6.33^°°°∗∗∗^
Serum 25(OH)D (ng/mL)				
low sun exposure	9.82 ± 4.57^§§§^	11.88 ± 8.12^∧^	9.96 ± 3.89	13.12 ± 8.71°°°
moderate sun exposure	13.23 ± 4.54^§§§^	14.66 ± 9.01	13.17 ± 4.11	16.77 ± 6.43^°°°∗^
good sun exposure	15.34 ± 4.43^§§§^	21.65 ± 8.84^∧∧∧§§§^	15.45 ± 3.98	24.55 ± 6.11^°°°∗∗^
Serum 25(OH)D (ng/mL)				
regular sunscreen use	10.14 ± 4.65^§§§^	17.13 ± 8.01^∧∧∧§§§^	10.29 ± 4.58	18.46 ± 7.55°°°
not regular sunscreen use	14.65 ± 3.99^§§§^	26.98 ± 8.77^∧∧∧§§§^	14.69 ± 4.88	26.03 ± 8.79°°°
Bone-specific alkaline phosphatase (U/L)	131.1 ± 37.3^§§§^	112.5 ± 30.6^∧∧∧§§§^	128.0 ± 34.9	121.7 ± 32.5^°∗∗∗^
Parathyroid hormone (pg/mL)	52.88 ± 33.41	43.01 ± 28.81^∧∧∧§§§^	54.54 ± 37.67	49.74 ± 33.71^*^

Arm A (*T*_0_ or *T*_1_) versus Arm B (*T*_0_ or *T*_1_):  ^*^ *P* < 0.05; ^**^ *P* < 0.005; ^***^ *P* < 0.001. Arm A (*T*_0_) versus Arm A (*T*_1_):  ^∧^ *P* < 0.05; ^∧∧^ *P* < 0.005; ^∧∧∧^ *P* < 0.001. Arm B (*T*_0_) versus Arm B (*T*_1_):  °*P* < 0.05; °°*P* < 0.005; °°°*P* < 0.001. Arm A (*T*_0_ or *T*_1_) versus Arm A (*T*_0_ or *T*_1_) or Arm B (*T*_0_ or *T*_1_) versus Arm B (*T*_0_ or *T*_1_):  ^§^ *P* < 0.05; ^§§^ *P* < 0.005; ^§§§^ *P* < 0.001.
